# Transmission of Resemblance from Parents to Children

**Published:** 1873-11

**Authors:** John Stockton-Hough


Laws of Transmission of Resemblance from Parents to their Children. — *John Stockton-Hough, M.D.*—Recapitulation: 1. In general, children of both sexes resemble their mother more than their father in physiognomy, habits,* constitution, and temperament.

2. Usually boys resemble their mother more than their father in physiognomy, habits, constitution, and temperament. In the same relationship girls resemble their father more than their mother.

3. As to whether there is any constant relationship between

*Dr. W. B. Carpenter, the distinguished physiologist, has recently written an able article "On the Hereditary Transmission of Acquired Physical Habits," in The Contemporary Review, April and May, 1873.

the physiognomical resemblance and a predisposition to the diseases of the person resembled, it is very difficult to decide from the data at hand, but it would appear from the few facts in which any observation were made in this direction, that there was a larger percentage of cases in which inherited diseases were exhibited where there was no resemblance than where there was such physiognomical similitude. In other words, children have resembled one parent in general physiognomy, while they have inherited the constitutional peculiarities and diseases of the other, more frequently than where they have derived both these conditions from (one) the same parent.

In general, then, hereditary and acquired diseases and defects are more likely to be transmitted to offspring of the sex in which they originated, and thereafter to be subject to the principle of sexual limitation, either directly from the parent to child, or by interrupted or atavic descent, from grandparent to grandchild.

Though sons are usually best able to follow the avocation of their fathers, it is undoubtedly true that men inherit the genius, talent, and intellectual excellence and morality of their mother or their mother's father, while daughters inherit the same qualities from their father or paternal grandmother.

4. *Females more fequently transmit* hereditary diseases and defects than males, though they *less frequently exhibit* them. Males less frequently transmit, and more frequently exhibit, inherited diseases and defects.

The reason that females do not exhibit hereditary disease as frequently as males, is because of a *higher degree of vitality* in them, which gives them greater power to restrain the appearance of the predisposition, and an *inferior degree of developmentat evolution*, retaining in their constitution as germs, what in men become fully developed diseases and defects.

